# Angiogenesis and vascular network of teratocarcinoma from embryonic stem cell transplant into seminiferous tubules

**DOI:** 10.1038/sj.bjc.6605125

**Published:** 2009-06-09

**Authors:** U Silván, J Arlucea, R Andrade, A Díez-Torre, M Silió, M A Konerding, J Aréchaga

**Affiliations:** 1 Laboratory of Stem Cells, Development and Cancer, Department of Cell Biology and Histology, Leioa, Vizcaya, Spain; 2 Biomedical Analytical and High Resolution Microscopy Facility, University of the Basque Country, E-48940 Leioa, Vizcaya, Spain; 3 Institute of Anatomy, Johannes Gutenberg University, D-55099 Mainz, Germany

**Keywords:** ES cell transplantation, embryonal carcinoma, teratocarcinoma, angiogenesis, vascular corrosion casting

## Abstract

**Background:**

Carcinoma *in situ* (CIS) of the testis is considered to be a precancerous germinal cell lesion, but the precise cellular and molecular mechanisms underlying transformation of CIS into invasive pluripotent cancer cells remain to be elucidated. Moreover, a satisfactory animal model for the experimental study of germinal tumours has not been developed to date.

METHODS: We have developed a tumour model that involves the microinjection of green fluorescent protein-labelled embryonic stem (ES) cells (which are functionally equivalent to CIS cells) into syngenic mouse seminiferous tubules, a unique cell microenvironment in which germinal cells mature and CIS arise. To characterise the vascularisation of teratocarcinomas, which arise after cell transplant, we used immunohistochemistry, together with a qualitative and quantitative analysis of scanning electron microscopy images of corrosion casting samples.

**Results:**

Embryonic stem cells transplanted into seminiferous tubules did not differentiate into germinal cells, but rather they behaved as invasive embryonal carcinoma (EC) stem cells. The vascular pattern of the experimental teratocarcinomas showed a highly disorganised architecture, and some of the neoplastic capillaries were derived, at least in part, from the original transplanted ES cells.

**Conclusion:**

The transplantation of pluripotent ES cells into seminiferous tubules efficiently recapitulates the early stages of development of teratocarcinomas. Consequently, this method constitutes a novel *in vivo* model to study the mechanisms of invasion and progression of experimental germinal tumours.

Tissue-specific stem cell origin of many tumours is a widely accepted theory (Aréchaga, 1993; Shipitsin and Polyak, 2008). Carcinoma *in situ* (CIS) of the testis is also recognised today as a precursor lesion for embryonal carcinoma (EC) and other germinal tumours (Skakkebaek, 1972). Moreover, EC cells, the cancer stem cell of testis teratocarcinomas, have the peculiarity of retaining the epiblast pluripotency of the original embryo during tumour invasiveness and differentiation (Kleinsmith and Pierce, 1964; Pierce, 1967; Aréchaga, 1993; Díez-Torre *et al*, 2004). Indeed, some embryo microenvironments have an extraordinary capacity to normalise their fate and differentiation (Brinster, 1974; Pierce *et al*, 1986; Aréchaga, 1998). These characteristics confer to EC cells the ability to give rise to neoplastic tissue components derived from the three germinal layers (teratoma). Interestingly enough, recent publications have also reported the high similarities between CIS cells and embryonic stem (ES) cells (Almstrup *et al*, 2006), and the easy transformation of ES to EC cells after prolonged adaptation to culture conditions (Andrews *et al*, 2005). Finally, it is important to keep in mind that cultured spermatogonial stem cells or modified ES cells, can be transplanted into the seminiferous tubules to restore fertility, representing an exciting possibility in the field of regenerative medicine (Ogawa *et al*, 1997; Brinster, 2007).

The present article is focused on examining the characteristics of the blood supply of a new tumour model based on the transplantation of ES cells into the mouse seminiferous tubules. In this regard, it is important to point out that the recruitment of new blood vessels, which carry oxygen, nutrients and other metabolites, is essential for the growth of cellular structures beyond a certain volume, because, among other things, diffusion of oxygen is limited to around 250 *μ*m inside organs and tissues (Folkman, 1985). If this process fails, cell growth within the tissue ceases and degenerates through an avascular necrotic process. The capacity of tumours to induce neovascularisation was reported over a century ago (Goldman, 1907), but it was not until the late sixties of the twentieth century when it was proposed that tumour cells secrete diffusible factors regulating vascular growth and proliferation (Greenblatt and Shubi, 1968). The role of tumour microenvironment is also an important matter to be considered for our correct understanding of the vascular supply of neoplasias (Nyberg *et al*, 2008).

## Materials and methods

### Animals

A total of 45 (12 control and 33 experimental) eight-week-old mice of the 129 P2 Ola/Hsd (Harlan Laboratories, Barcelona, Spain) strain were used. They were bred under a 12-h light–darkness cycle and had free access to water and food.

### Cell line

Transplanted ES cells belong to the AB1 cell line (McMahon and Bradley, 1990) that has the same genetic background of the recipient animals. For fluorescent labelling, cells were transfected with the pEGFP plasmid (Clontech, Saint-Germain-en-Laye, France; Cat. 6076-1) using the cationic lipid Lipofectamine 2000 (Invitrogen, Carlsbad, CA, USA; Cat. 11668-027). A fluorescent cell colony was selected from the resulting culture and expanded in the presence of the selection antibiotic G418.

### Cellular transplantation

**Figure 1:**
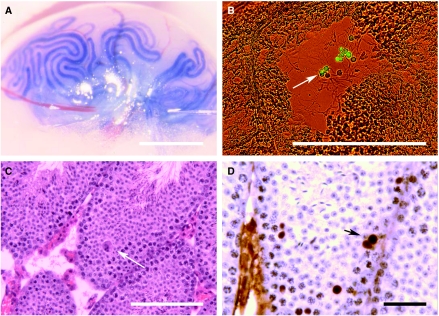
(**A**) Cell microinjection into the seminiferous tubules. The blue dye used to monitor the precision of transplantation can be seen filling the seminiferous tubule network. (**B**) Shortly after transplantation, fluorescent cells (arrow) could be found in the lumen of the seminiferous tubules in cryostat sections. (**C**) At 36 h after transplantation, some of the AB1^GFP^ cells were found integrated in the seminiferous epithelium (arrow). (**D**) These cells were recognised with anti-GFP-specific antibodies (arrows). Bars represent 250 *μ*m in image A and 100 *μ*m in images **B**, **C** and 50 *μ*m in image **D**.

Cell transplants into the seminiferous tubules were carried out following the Ogawa *et al* method (Ogawa *et al*, 1997) (Figure 1A). Briefly, an AB1^GFP^ cell suspension (2 × 10^6^ cells ml^−1^), containing the visual tracer bromophenol blue at a non-toxic concentration, was microinjected using sharpened borosilicate capillaries into seminiferous tubules of Avertin-anaesthetised mice. The injection pressure was generated by an Eppendorf air injector and the process was carried out under a MZ Apo stereomicroscope (Leica Microsystems, Wetzlar, Germany) with coaxial illumination. Transplantation was considered satisfactory if most of the surface tubules were filled by the cell suspension and no dye was seen in the stroma. A total of 35 testes were correctly transplanted, of which 17 (49%) developed a tumour. Mice were killed 5 weeks after transplantation. All the experiments were carried out *in vivo* after approval from the University of the Basque Country Ethical Committee and were compliant with the UKCCCR Guidelines for the Welfare of Animals in Experimental Neoplasias (Workman *et al*, 1998).

### Corrosion casting

Mice were anaesthetised with pentobarbital (0.1 ml, Narcoren, Merial, Germany) and aorta was catheterised with an olive-tipped needle. The whole vascular tree was washed out with physiological buffered saline including heparin (Liquemin 5000 IE, Roche Farma España, Madrid, Spain). Next, 10 ml of 2.5% glutaraldehyde in Ringer's saline solution and, subsequently, 40 ml of Mercox CL-2B resin (Vilene Med Co., Tokyo Japan) diluted with 20% methylmethacrylate monomers (Merck Darmstadt, Germany) were perfused. Then, the entire animal was immersed for 1 h in a 40°C water bath to facilitate resin polymerisation. Normal size and tumoral testes were removed and immersed in a 5% KOH solution to digest all the tissues around the vessel casts. One week later the specimens were dehydrated, mounted on conductive stubs and coated with gold in an SCD 040 sputter-coater (BAL-TEC AG, Leica Microsystems). The specimens were visualised under a Philips XL30 scanning electron microscope (Philips, Eindhoven, The Netherlands), and between 20 and 30 stereo pair images with a 6° tilt angle were recorded per sample.

### Morphometry and statistical analysis

In order to carry out the morphometric study of vascular corrosion casts, we used 13 tumour and 11 normal testes. Pairs of stereo images were analysed with the image analysis program, Kontron KS 300 (Carl Zeiss Vision, Eching, Germany), to calculate the parameters associated with microvascular architecture, such as the intervascular and interbranching distances and the vascular diameters. For details of reconstruction and calculations see Malkusch *et al* (1995). Statistical analyses and graphic displays were performed using Sigmaplot and SPSS software (Chicago, IL, USA). Statistical significance was evaluated using the Mann–Whitney and Student's *t*-tests.

### Immunohistochemistry

Rat primary antibodies (Santa Cruz Biotechnology Inc, Santa Cruz, CA, USA) against CD31 and VEGF-R2 diluted 1 : 100 in saline buffer were used to detect endothelial cell markers. Cryostat sections were incubated for 1 h with blocking serum (5% fetal calf serum in saline buffer) and, later, overnight with the primary antibody at 4°C. Histological sections were incubated for 1 h with secondary fluorescent antibody (goat anti-rat Texas Red labeled, Santa Cruz Biotechnology Inc, diluted 1 : 400). Results were observed and photographed using a confocal scanning microscope Fluoview FV500 (Olympus, Tokyo, Japan).

## Results

Undifferentiated AB1^GFP^ ES cells microinjected into the specific microenvironment of mouse seminiferous tubules (Figure 1A) did not differentiate into germinal or Sertoli cells, but rather into teratocarcinomas. Thus, this ES cell transplant into mouse seminiferous tubules mimics the niche of pre-invasive germinal neoplasias of the testis (CIS), its earliest invasion stages and the histopathology of teratocarcinomas (Pierce, 1967; Damjanov *et al*, 1983). The presence of fluorescent ES cells inside the seminiferous tubules, and not in the testis stroma, was confirmed after their histological study of the injected testes shortly after transplantation (Figure 1B). Thus, 36 hours after transplantation, transplanted labelled ES cells were found integrated into the seminiferous epithelium (Figure 1C), as evidenced by using green fluorescent protein (GFP)-specific antibodies (Figure 1D).

**Figure 2:**
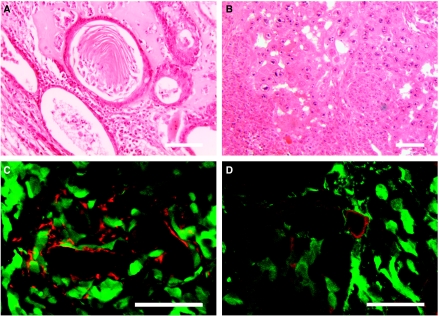
Histology and histochemistry of experimental teratocarcinoma tumours. (**A**) Epidermoid and osteoid differentiation. (**B**) Undifferentiated embryonal carcinoma cells. Capillary-like structures positive for endothelial cell markers CD31 (in **C**) and VEGF-R2 (in **D**) co-localize with labelled cells (green fluorescence) derived from transplanted AB1^GFP^ ES cells. Bars represent 50 *μ*m.

A total of 5 weeks after transplantation, a teratocarcinoma was formed in 49% of injected testes (*N*=35) and the tumours showed the characteristic structure of the spontaneous EC of the testis (Figures 2A and B). With regard to tumour vascularisation, confocal microscopy histochemistry of endothelial cells of tumour capillary vessels exhibited CD31 and VEGF-R2 immunoreactivity and, simultaneously, exhibited GFP fluorescence, showing their origin from the transplanted fluorescent ES cells (Figures 2C and D). These findings indicate that transplanted ES cells have the capacity to differentiate into neoplastic capillaries.

**Figure 3:**
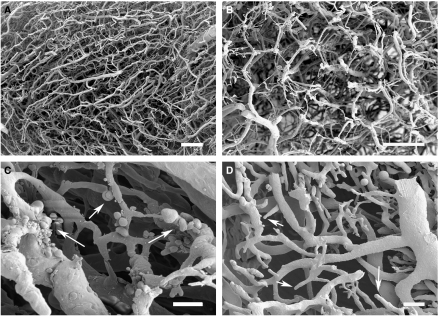
Testis vascular casts of the longitudinal capillaries (**A**), which run parallel to seminiferous tubules, and of the transverse capillaries (**B**) that connect longitudinal capillaries every 100–200 *μ*m and surround the seminiferous tubules, exhibiting the appearance of a honeycomb. Vascular teratocarcinoma network with resin extravasations (**C**) and blind endings (**D**) marked with arrows. Bars represent 100 *μ*m in A and B, 50 *μ*m in **C** and 250 *μ*m in **D**.

Scanning electron microscopy of vascular corrosion casting samples of tumours showed capillaries with irregular diameters, chaotic hierarchy and several blind-endings similar to those found in lymphatic vessels. Resin extravasations and vascular compressions were also frequent. These characteristics are typical of an immature vascularisation pattern and differ significantly from those observed in normal testis histology (Figure 3). These differences were quantified by measuring three morphometric parameters: (a) intervascular distance, (b) interbranching distance and (c) vascular diameter.

**Figure 4:**
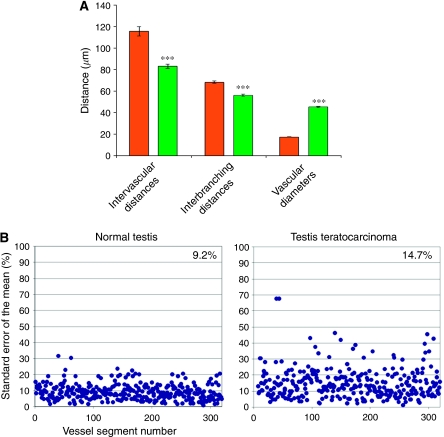
(**A**) Comparison of the means from the measured parameters in normal testis capillaries (in dark grey) and teratocarcinoma capillaries (in green). Statistically significant differences are indicated (^***^P<0.05). (**B**) Spectra of vessel diameters in healthy testis and teratocarcinoma. The percent deviations from the mean diameter of four or five distinct points were calculated for each vessel segment (300 vessel segments were measured). Teratocarcinoma samples seem to be characterized by a larger dispersion of these values, indicating a more chaotic vascular bed, probably because of the imbalance of vasculogenesis regulation. Variation of the mean vessel diameter is shown in the upper right corners, and illustrates the much greater dispersion in vascular size in tumours, in comparison with normal tissues.

The mean values and the standard deviation of the measured parameters in both normal testis and experimental teratocarcinoma are represented in Figure 4. Maximal and minimal values are shown in Table 1, together with the number of measurements and the mean values.

As shown in Figure 4A, the mean intervascular distance was 15% shorter in teratocarcinomas than in healthy testis and this difference was statistically significant. Moreover, we observed that the highest and lowest values of the mean were found in the tumour casts (Table 1), an indication of the disorganisation of tumour vascular network. Neoplastic and normal tissue vascularisation showed also differences in their interbranching distances (Figure 4A) and these were both, larger and of wider range, in the tumour casts in comparison with the healthy organ.

The characteristic branching angles of the ladder-like pattern of testis vasculature were completely lost in the tumours (Figure 3) and the mean vascular diameter of its vessels (Figure 4A) was 130% larger than in normal testis. These data, together with the shorter intervascular distances, which are indicative of a higher vascular density, show that the volume occupied by vessels was much larger in tumours than in normal testis. Once again, the widest and the narrowest capillaries correspond to the tumour vasculature casts (Table 1). Figure 4B illustrates the standard error of the mean (s.e.m.) of the diameter for each segment in the healthy organ and tumour casts; normal testis was associated with a s.e.m. value of 9.2%, whereas the s.e.m. for tumour vasculature was 14.7%. It is likely that the greater irregularity in vascular diameters is associated with chaotic blood flow within the disorganized tumour tissue.

## Discussion

The incidence of testicular germ cell tumours (TGCTs) in young males has risen during last decades (Cooper *et al*, 2008) and this increase seems to be related to the environmental factors, although no consensus has yet been reached regarding the precise aetiology (McGlynn *et al*, 2005; Shah *et al*, 2007). In contrast, these tumours are very uncommon among non-human mammals. In this regard, Murine gene deletions, that promote an increase in the incidence of testis teratomas in 30% of carriers, have been reported a long time ago (Stevens, 1973), but most of these tumours are differentiated (benign teratomas) and, for that reason, not adequate as a model for studying human testis teratomas, which are usually malignant. An important fact to be underlined in this regard is the phenotypic similarities between ES cells and testis CIS cells (Andrews *et al*, 2005; Almstrup *et al*, 2006). For this reason, ES cell microinjection inside mouse seminiferous tubules represents a model with high potential utility, because it mimics, in many ways, the pre-invasive state of TGCTs.

In order to identify structures derived from microinjected cells, we labelled Murine ES cells with GFP to facilitate their tracking after transplantation into the seminiferous tubules. We found that most of the neoplastic tissue comes from the AB1^GFP^ cells, although fluorescence intensity was found to be quite heterogeneous. Nevertheless, it should be noted that the intensity of GFP fluorescence labelling could depend on oxygen tension (Takahashi *et al*, 2006), indicating that the heterogeneity of fluorescence intensity may be due to the variable oxygen concentration inside the tumour.

In this study, we focused on tumour vascularisation because of its importance for neoplastic invasion and metastasis of germinal tumours. In this regard, two specific mechanisms of tumour vessel growth have been identified to date, namely angiogenesis and vasculogenesis (Carmeliet and Jain, 2000; Velazquez, 2007). In our experimental model, we found evidence for another possible mechanism, that of the genesis of neoplastic capillaries from the highly plastic transplanted ES cells. Thus, some of the endothelial cells of the experimental teratocarcinoma were found to express the endothelial cell markers CD31 or VEGF-R2 and simultaneously exhibited green fluorescence, indicating their origin from the transplanted AB1^GFP^ cells. This ability of cancer stem cells to give rise to capillary endothelia has been reported for other tumours, such as lymphoma (Streubel *et al*, 2004), myeloma (Rigolin *et al*, 2006) and neuroblastoma (Pezzolo *et al*, 2007). In our experimental teratomas, some of the cells covering the lumen of the blood vessels were not found to express the above mentioned endothelial markers, but such a ‘vessel mosaic’ has already been described in other tumours (di Tomaso *et al*, 2005).

Earlier corrosion casting works have shown that branching angles and interbranching distances are specific and characteristic for each tissue including tumours (Konerding *et al*, 1995, 1999). In our case, the vascular branching and bifurcation capillary pattern of the normal testis totally disappeared and the examined specimens exhibited features similar to an immature vascular network with heterogeneous vessel densities, frequent blind-ending, resin extravasations and vascular compressions (Jain, 1988). In this regard, intervascular distances were considered to be a parameter indicative of vascular density, similar to microvessel density used in earlier bidimensional studies. Capillaries in experimental teratocarcinomas were about 15% closer than in the healthy tissue. This increase in vascular density is common to most tumours, although there are some exceptions; pituitary tumours, for example, show higher intercapillary distances than the equivalent healthy tissue (Turner *et al*, 2000). In some tumours, such as retinoblastoma or nephroblastoma, vascular density is used as a criterion for prognosis (Abramson *et al*, 2003; Rössler *et al*, 2004) because of the higher risk of metastasis and better access of the tumour cells to nutrients and oxygen. Finally, it is important to point out that the mean vascular diameter of teratocarcinoma is about 2.4 times wider than in the normal testis. This broader distribution of the diameters and the higher values of the irregularities observed in different vascular segments may be because of altered blood flow and to the high pressure of the tumour stroma. Both characteristics would also cause a higher vascular permeability and therefore worse clinical prognosis.

Recent advances in stem cell research, such as the possibility of obtaining pluripotent stem cells from adult tissues (Takahashi and Yamanaka, 2006) and their capacity to differentiate *in vitro* into almost all kinds of tissues, have generated many therapeutic expectations (Atala, 2007; Fish and Grupp, 2008). However, as this work shows, the risk of generating a tumour after these transplants is a real possibility, especially when employing cultured stem cells. This suggests that stem cells in the adult mammals may be under physiological or epigenetic controls in order to be organ-specific; this may be a defense mechanism to avoid defective differentiation and tumour formation.

Nowadays, treatment of germinal tumours has a high success rate (Gerl *et al*, 1996; Bosl, 1999). However, early tumour detection and identification of the causes underlying their increased incidence among young men remain to be determined, as do the cellular and molecular mechanisms of CIS to EC cell transformation. We have developed a tumour model that can facilitate the study of the pre-invasive state of malignant teratomas, based on the tumorigenic potential of cultured ES cells. Tumours arising from the transplantation of AB1^GFP^ cells recapitulate the phenotypic and molecular features of spontaneous human teratocarcinomas, substantiates the potential utility of this assay for the screening of novel therapeutic strategies, particularly involving the inhibition of angiogenesis and metastasis.

## Figures and Tables

**Table 1:** Vascular parameters in healthy and tumoral testis (*N*: registered vascular measurements)

		**Normal testis**	**Teratocarcinoma**
Intervascular distances	N	1931	2242
	Mean	68.35	53.15
	s.e.m.	0.90	0.85
	Higher value	284.19	324.8
	Lower value	8.02	4.03
Interbranching distances	N	569	870
	Mean	83.10	100
	s.e.m.	1.88	3.17
	Higher value	339.6	984.5
	Lower value	11.67	2.34
Vascular diameter	N	1840	2576
	Mean	17.36	40.85
	s.e.m.	0.18	0.5
	Higher value	71.44	272.5
	Lower value	4.91	3.76
